# Healthy Gut, Healthy Bones: Targeting the Gut Microbiome to Promote Bone Health

**DOI:** 10.3389/fendo.2020.620466

**Published:** 2021-02-19

**Authors:** Olivia D. Cooney, Prabhakar R. Nagareddy, Andrew J. Murphy, Man K. S. Lee

**Affiliations:** ^1^ Haematopoiesis and Leukocyte Biology, Baker Heart and Diabetes Institute, Melbourne, VIC, Australia; ^2^ Department of Diabetes, Monash University, Melbourne, VIC, Australia; ^3^ Division of Cardiac Surgery, Department of Surgery, Ohio State University, Columbus, OH, United States; ^4^ Department of Cardiometabolic Health, The University of Melbourne, Melbourne, VIC, Australia

**Keywords:** gut, microbiome, bone, Th17 & Tregs cells, short chain fatty acid

## Abstract

Over the past decade, the use of probiotics to modify the gut microbiome has become a public spotlight in reducing the severity of a number of chronic diseases such as autoimmune disease, diabetes, cancer and cardiovascular disease. Recently, the gut microbiome has been shown to play an important role in regulating bone mass. Therefore, targeting the gut microbiome may be a potential alternative avenue for those with osteopenia or osteoporosis. In this mini-review, we take the opportunity to delve into how the different components of the gut work together and how the gut-related diseases impact on bone health.

## Introduction

Over a century ago, Metchnikoff had discovered that complex living organisms, now known as microbes, were living symbiotically within the human gut (1). However, their contribution to human health and disease remained understudied. Fast-forward to the past decade and we have seen the gut microbiome taking center stage in various diseases. This has been due to the advance in cutting-edge technologies such as 16S ribosomal RNA sequencing and shotgun metagenomics. Numerous pre-clinical studies now demonstrate that the diversity of the gut microbiome influences a wide range of diseases including autoimmune disorders (2–4), diabetes (5, 6), obesity (7, 8), cardiovascular disease (9, 10), and cancer (11, 12). Interestingly, a growing body of evidence suggests that reintroducing ‘good’ bacteria to the microbiome in the form of probiotics can dampen the severity of disease (13, 14). Recently, a new interdisciplinary field bridging the study of gut microbiome and bone biology, known as ‘osteomicrobiology’ has emerged. Over the past couple of decades, various groups have documented the influence of the gut microbiome on bone health and disease.

The skeletal bone is an essential organ that provides the human body with both structural (mobility and support) and a reservoir (storage for minerals such as calcium and phosphorus) function. Bones are composed of minerals deposited around protein, which allows the bone to absorb without breaking (15). The minerals within the bone consist mostly of calcium and phosphorus, important for providing ‘hardness’ to the bone. On the other hand, proteins, which are made up of a dense network of collagen are important for adding ‘softness’ to the bone. Together, they form a scaffold allowing the bone to sustain some degree of mechanical pressure without easily breaking. During childhood to adolescence, the bone predominantly undergoes a process known as ‘modeling’ where new bones are formed at one site of the bone while on the other side old bones are removed, allowing the bone to change its size/shape. Modeling in humans typically reaches its peak by 20-30 years of age, after which, a different process known as ‘remodeling’ occurs (16). This is when old bones are removed and replaced with new bones, which is important especially when bone fractures occur or when old bones become brittle and needs replacing. In addition, remodeling can also be activated when the body is deficient or in need of calcium and phosphorus for other cellular or tissue functions.

Bones are formed by non-hematopoietic cells known as osteoblasts (OBs), which are derived from stromal cells within the bone marrow (BM). OBs produce collagen that forms a scaffold for calcium and phosphorus to be deposited into, thus laying down new bone (17). On the other hand, osteoclasts (OCs) resorb bone and are derived from hematopoietic stem cells also located within the BM. OCs adhere and secrete hydrogen ions to the surface of the bone, which dissolves and releases the mineral deposits from the bone. Consequently, constant interaction between OBs and OCs are essential during bone remodeling to maintain bone homeostasis (18). Unfortunately, when bone homeostasis is not maintained, osteopenia or debilitating bone diseases such as osteoporosis can occur. This is mainly due to an over activation of OC activity, which degrades more bone than OBs can form new bones, or, when OB activity is inhibited. Osteoporosis is a global health crisis and primarily occurs within the aging population worldwide (19). As such, the need for deeper understanding on how to maintain bone health and how to treat those with osteopenia is vital as the human lifespan continues to increase thanks to modern medicine.

While the lion’s share of the research on bone biology has focused on therapeutics that can directly target the bone to prevent or treat osteoporosis, such as bisphosphonates, it is becoming apparent that the gut may have indirect effects in maintaining bone health. Thus, targeting the gut may be an attractive alternative therapy. In this brief review, we briefly discuss the importance of the gut in maintaining body homeostasis. Next, we discuss in more detail the interactions between the gut and the bone by exploring emerging mechanisms that have come to light in recent years.

## The Gut Microbiota Can Influence Intestinal Permeability in Gut-Related Diseases

**Figure 1:**
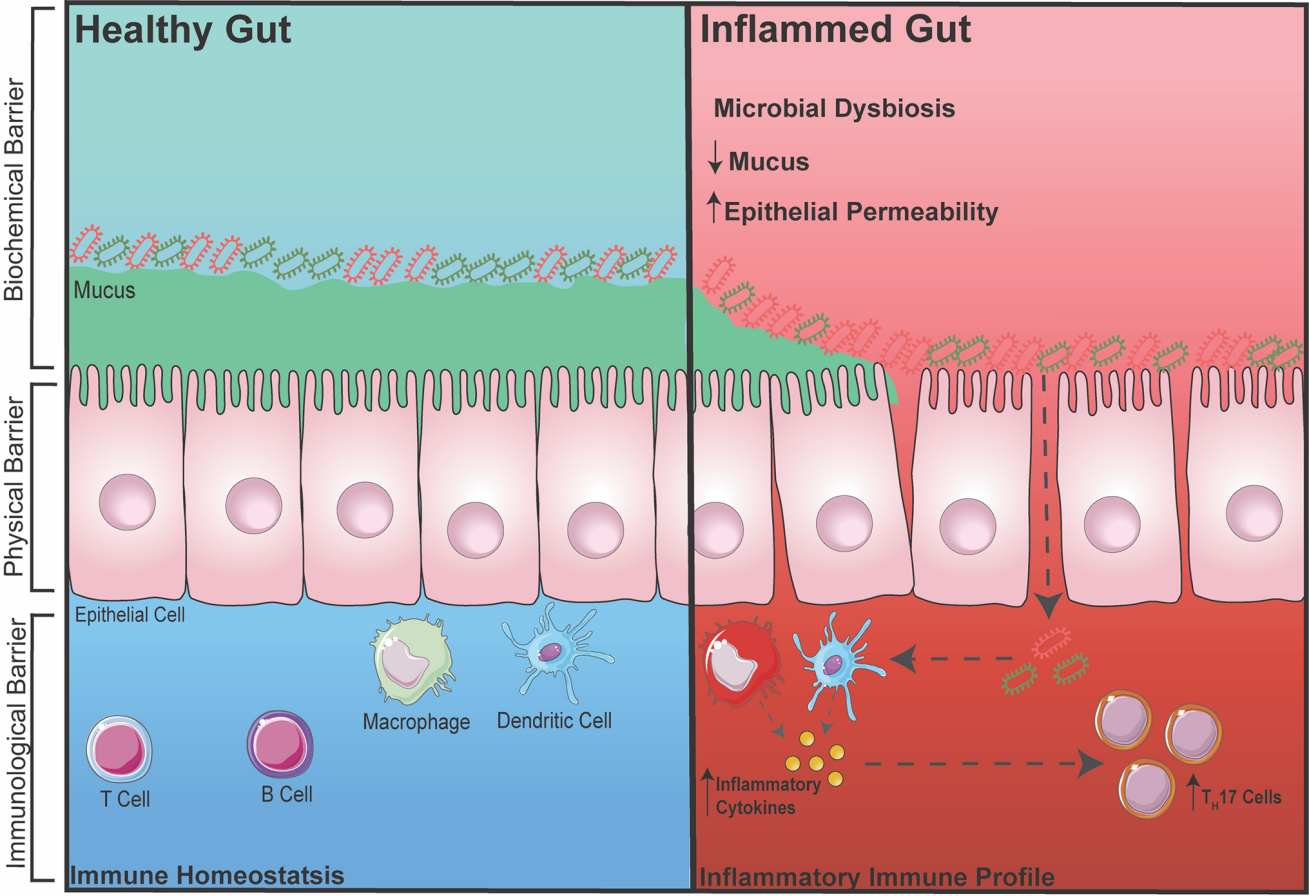
Healthy vs. Inflamed Gut During intestinal homeostasis (BLUE) the biochemical barrier contains a mucus layer that helps to prevent GM from contacting the epithelial layer. The physical barrier consists of tightly-packed single layer of epithelial cells connected by intercellular junctions. The immunological barrier consists of innate and adaptive immune cells that help to surveillance the gut for any foreign entry of GM (gram positive/negative bacteria). During gut-related inflammation (RED), the physical barrier can become compromised, increasing intestinal permeability, making it susceptible for unwanted GM to pass through and activating an immune response.

The gut is one of the largest organs in the body. As part of the digestive tract, its main goal is to absorb vital nutrients into the bloodstream in order to maintain energy for cellular and body functions. However, in addition to resident gut microbiota (GM), the food and drinks we consume consist of many foreign pathogens/toxins; therefore, without a defense barrier within the gut, critical and complex functions in the body becomes compromised. Thus, to protect the body, there are multiple defense barriers inside the gut. One of the most important barriers of the gut is a single layer of tightly-packed intestinal epithelial cells (IECs), which act as a physical barrier that separates the outside environment from the inside ‘sterile’ organs of the body (20, 21). These IECs are connected *via* intercellular junctions composed of three junctions; tight junctions (TJs), adherens junctions (AJs), and desmosomes. These junctions work together to assist with selective transport of ions across the epithelial layer and to also protect the integrity of the epithelial layer. To further aid the integrity of IECs, specialized cells among the epithelial layer known as goblet cells secrete mucin to add and maintain a layer of mucus (22). This acts as a biochemical barrier preventing GM and pathogens from directly interacting with the epithelium. The mucus layer is subdivided into an inner and outer mucus layer, in which the GM exist in the outer layer consisting of ~10^4^ bacterium from over 100 species resides (23). Despite the detrimental effects the GM potentially poses to body, when kept at homeostasis, the GM plays an essential role in gut functionality by aiding the digestion of foods that the gut itself cannot process (23). Finally, below the epithelium is a host of immune cells, which act as a third line of defense to prevent the GM from entering the systemic circulation. These gut immune cells, including macrophages, dendritic cells, T cells and B cells, are housed within gut associated lymphoid tissues such as Peyers Patches or scattered throughout the lamina propria (24, 25) (**Figure 1**).

During inflammatory disease states, the intestinal barrier can become compromised, increasing intestinal permeability. This makes the body susceptible to foreign particles or GM entering into circulation thereby exacerbating inflammation (26–28). This term is also commonly known as a ‘leaky gut’. It was recently demonstrated in a pre-clinical model of RA that the TJ protein, zonula occludens (ZO)-1, which is known to stimulate the opening of TJs, was significantly increased, leading to inflammation. When ZO-1 was inhibited using a zonulin antagonist, inflammation in RA was markedly dampened (29). In addition, increased intestinal permeability has also been associated with inflammatory bowel disease (IBD) due to dysregulation of the pre-dominant AJ protein, E-cadherin (30). Metabolic diseases such as diabetes and obesity have also been linked to increased intestinal permeability, which again is involved in further increasing inflammation (31, 32). Besides direct dysregulation of intercellular junctions, a reduction in the protective mucus layer secreted by goblet cells is also known to cause a breakdown of the barrier integrity allowing for GM to interact and pass through the IECs, which is one of the hallmarks of IBD (33). Interestingly, it was recently shown that the diversity of GM can also affect the integrity of the intestinal barrier and inflammation. With accumulating evidence over the years, restoring or balancing the GM has become an attractive therapeutic avenue in preventing increased intestinal permeability and dampening inflammation. Reintroducing ‘good’ bacteria by supplementing diets with probiotics has been heavily investigated in both mice and human, in which the supplementation of probiotics has been shown to restore the GM and reduce disease severity. For example, increased dietary choline (9) (obtained from foods such as eggs) and carnitine (34) (from red meats) can cause atherosclerotic-cardiovascular disease *via* the gut microbiota-derived metabolite TMA which converts into TMAO in the liver. By administrating *Lactobacillus*, TMAO levels were reduced, thereby reducing the development of atherosclerosis (35). Furthermore, diabetes has been shown to shift the abundance of two dominating bacterial phyla, Firmicutes and Bacteroidetes that leads to increased disease severity. Supplementation with *Lactobacillus* was shown to reduce inflammation (36, 37). However, with a biased focus now on the effects of probiotics and how it may reduce inflammation, many studies now fail to also investigate whether administering probiotics reduces inflammation due to a restoration in intestinal permeability and immune cells in the gut. Thus, it would be beneficial to measure all three components (GM, immune cell, and intestinal permeability) when intervening with probiotics in gut-related diseases.

## Potential Mechanisms of How the Gut Microbiota Influences Bone Mass

In the past two decades, gut-related inflammatory diseases have been linked to a decrease in bone mass, suggesting that the gut may be interlinked with the bone. Recently, it was shown that people with osteoporosis had significantly higher microbiome diversity compared to healthy individuals, specifically in the abundance of Firmicutes (38). In line with this, another clinical study comparing healthy individuals, people with osteopenia and patients with osteoporosis, showed that the severity of bone loss was correlated to the diversity of the gut microbiome (39). In addition, a clinical study conducted in elderly women with low bone mineral density proposed that restoring the microbiome with supplementation of probiotics reduced bone loss (40). Although, the use of probiotics may be a viable therapeutic option, more investigation on how they influence bone biology is warranted for any major changes for treating bone defects. For example, in a recent clinical trial, it was shown that probiotics had no effect on hip bone mineral density, but rather showed a reduction in femoral neck bone mineral density (41). Therefore, studies in pre-clinical mouse models are critically important to directly test how changes in the GM mechanistically impacts the bone.

The Ohloson group was the first to discover in mouse models that germ-free (GF) mice had significantly increased trabecular bone volume compared to conventionally raised (CONV-R) mice (42). In support of this, depletion of the gut microbiota, through antibiotic administration, has also been shown to restore bone mass (43, 44). Despite these findings, it is still unknown exactly why total deletion of GM would be beneficial for bone health. One reason may be that there is a slow or oscillating penetrance of microbial products through the gut that through any number of pathways result in the activation of OCs or suppress OBs. Whether this occurs during development when the gut is not fully formed to prevent unwanted bacterial translocation into bloodstream of the body is also a potential mechanism. However, once the body (and gut) is developed, the GM is clearly beneficial for a number of biological functions central to human health. The symbiotic relationship between the gut and the microbiome is important in the absorption of nutrients that the gut itself cannot process. The GM is also a source for vitamin K2, which is required for the function of osteocalcin and can influence bone formation by stimulating OBs (45, 46). Moreover, studies have shown that decreased levels of vitamin K2 due to antibiotic-induced microbiome depletion is associated with a reduction in osteocalcin and bone strength in mice (47). Based on the relationship between the gut and bone, re-introducing beneficial strains of bacteria in the form of probiotics has recently garnered interest in the bone field. Pre-clinical mouse studies have found that administering probiotics such as VSL#3 and bacterial strain *Lactobacillus rhamnosus* GG can be beneficial for bone health *via* restoring GM and intestinal permeability (48). In estrogen deficient mice, in which estrogen dampens cytokines involved in stimulating osteoclastogenesis and bone loss (49, 50), it was found that the administration of *Lactobacillus reuteri* protected against bone loss. Moreover, other studies have demonstrated that in mice with glucocorticoid-induced microbial dysbiosis or post-antibiotic-induced gut dysbiosis, supplementation of *Lactobacillus reuteri* could dampen trabecular bone loss by reducing gut dysbiosis and intestinal barrier dysfunction (51–53).

**Figure 2:**
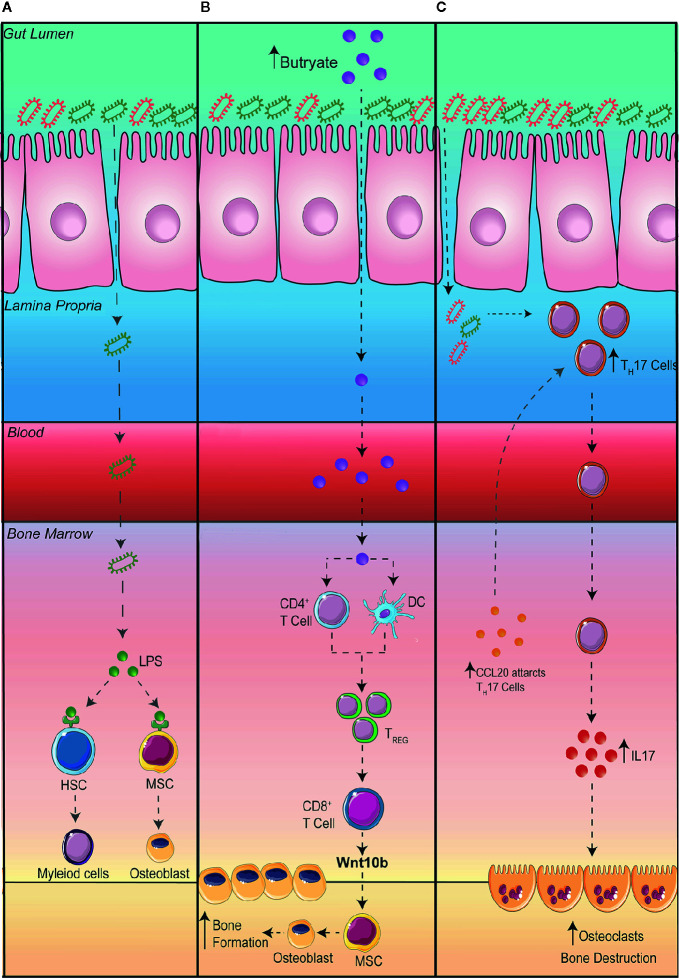
Potential mechanisms connecting the gut to the bone. **(A)** A leaky gut can result in bacteria translocating to other organs such as the bone where lipopolysaccharides (LPS) are recognized by toll-like receptor (TLR) 4 on hematopoietic stem cells (HSC) and mesenchymal stromal cells (MSC). **(B)** Increasing the production of butyrate in the intestine promotes bone formation via an increase in the differentiation of regulatory T cells (Treg). Tregs stimulate CD8^+^ T cells to secrete Wnt10b promoting the differentiation of osteoblasts and bone formation. **(C)** An expansion of T Helper (T_H_) 17 cells in the gut can result in the migration of T_H_17 cells to the bone. Additionally, the upregulation of the chemoattractant CCL20 in the bone marrow can aid the migration of intestinal T_H_17 cells to the bone where the production of interleukin (IL) 17 can promote the differentiation of osteoclasts thereby promoting bone destruction.

Despite the potential therapeutic approach in using probiotics to reduce intestinal permeability and bone loss, the question still remains as to how exactly changes in the GM influences bone homeostasis. Obviously, increased intestinal permeability could result in the translocation of bacteria or its microbial products to the bone, increasing and prolonging osteoclastogenesis. In addition, to prevent systemic infection, immune cells within the bone and in other organs express toll-like receptors (TLRs) which recognize pathogen associated molecular patterns. TLR4, one of the most well studied TLRs, is highly expressed on immune cells and is activated by lipopolysaccharides (LPS) and damage-associated molecular pattern ligands. Once activated, TLR4 promotes innate immune responses and the production of inflammatory cytokines. In addition, an increase in inflammatory cytokines, particularly IL-23 can promote the maturation of pathogenic T_H_17 cells in the BM, which in turn can stimulate osteoclastogenesis, leading to increased bone loss (54). TLR4 has also been shown to be expressed on mesenchymal stromal cells (MSCs) which plays a critical role in bone formation (55–57). The activation of TLR4 on MSCs has been shown to promote the differentiation of osteoblasts through Wnt3a and Wnt5a signaling (55). While osteoblasts promote bone formation, some studies have suggested that when stimulated by LPS they can promote the differentiation of OCs and bone degradation (**Figure 2A**) (56, 57).

Metabolites, in particular short chain fatty acids (SCFA), produced by GM can play essential roles in regulating immune responses (58, 59). Interestingly SCFA have been linked to improving bone health (60–62). A series of studies by the Pacifici group explored the cross talk between the gut and the bone. Firstly, they demonstrated that promoting the production of butyrate with the administration of lactobacillus or directly supplementing mice with butyrate promoted bone formation *via* an increase in the expression of osteogenic Wnt ligand Wnt10b from Treg cells in the BM. This activates the Wnt signaling pathway in osteoblasts to increase bone formation (61). In a separate study, to further elucidate how the gut communicates with the bone, the Pacifici group investigated how butyrate influences bone formation in response to parathyroid hormone treatment. To determine whether butyrate signals *via* T cells in the BM, they adoptively transfer splenic T cells from *Gpr43^+/+^* and *Gpr43^-/-^* mice into *Tcb^-/-^* mice, a mouse model that lacks T cells (62). After donor T cell reconstitution, they supplemented mice with butyrate in model that recapitulates bone loss *via* administering parathyroid hormone inhibitors (iPTHx) and confirm that butyrate does not directly signal through GPR43 on T cells to increase bone formation. Next, they hypothesized that butyrate may be acting on GPR43 on myeloid cells particularly dendritic cells. To do this, they co-cultured BM isolated dendritic cells from *Gpr43^+/+^* or *Gpr43^-/-^* mice with WT T cells and discovered that GPR43 on dendritic cells was required to differentiate T cells into Treg cells, which then go on to expressing Wnt10b to stimulate bone formation (**Figure 2B**).

The dysregulation of gut homeostasis can result in an inflammatory immune phenotype. This includes an increase in interleukin IL-17 producing T_H_17 cells. Studies have shown that an increase in T_H_17 cells and IL-17 in the BM promotes bone degradation by stimulating the differentiation of OCs in the BM (63, 64). The Pacific group investigating the role intestinal immune cells play in bone remodeling in the setting of hyperparathyroidism (65). They demonstrated that in mice with microbiomes enriched with segmented filamentous bacteria, parathyroid hormones expanded the population of gut T_H_17 cells which egressed out of the gut, into circulation, migrating into the BM to cause bone degradation (65). To show egress of intestinal T_H_17 cells, they inhibited sphingosine 1 phosphate (S1P) receptor-1 with an FTY720 antagonist, which prevents the egress of lymphocytes from the mesenteric lymph nodes and showed a decrease in BM T_H_17 cells and bone degradation. Furthermore, to show specifically the importance of T_H_17 cell migration into the BM, they showed that the chemoattractant CCL20 was upregulated in the BM, which acts to guide T_H_17 cells. When they administered neutralizing anti-CCL20 antibody, the number of intestinal T_H_17 cells were unaltered but it prevented the increase in T_H_17 cells in the BM as well as a reduction in bone loss. Furthermore, they also differentiated T_H_17 cells from isolated splenic T cells from IL-17A-eGFP reporter mice, transplanted into recipient mice and counted GFP+T_H_17 cells after inducing bone loss with infusion of parathyroid hormones (**Figure 2C**).

## Concluding Remarks

The inter-disciplinary roles between gut and bone has increasingly garnered attention in those in the field of bone biology. From knowledge gathered so far, it seems that restoring the balancing in GM using probiotics or prebiotics may be beneficial in restoring bone health. However, there are still many questions to be answered before the use of probiotics should be recommended to the aging community who are more susceptible to osteopenia. This will require a multi-disciplinary approach where microbiologists, immunologists, gastroenterologists, computational scientists, and the respective disease experts to work together to find the best approach to cure gut-related diseases.

## Author Contributions

All authors listed have made a substantial, direct, and intellectual contribution to the work and approved it for publication.

## Funding

AM is supported by a Centenary Award from CSL. OC is supported by an RTF scholarship from Monash University and also a Bright Sparks award from the Baker Heart and Diabetes Institute.

## Conflict of Interest

The authors declare that the research was conducted in the absence of any commercial or financial relationships that could be construed as a potential conflict of interest.
